# Supplementary material for: Patterns of Intron Gain and Loss in Fungi
Source: PLoS Biol. 2004 Nov 30;2(12):e422. doi: 10.1371/journal.pbio.0020422 (PMC532390; doi:10.1371/journal.pbio.0020422)
Supplement: Table S1 — Also available at http://genes.mit.edu/NielsenEtAl/. (4.3 MB ZIP). [file pbio.0020422.st001.zip › NielsenEtAl/html/428.html]

AN5719.1.NCU08960.1.MG06185.1.FG10368.1


```
 CLUSTAL W (1.82) Multiple Sequence Alignments - Introns Inserted


Sequence 1: NCU08960.1	109 aa
Sequence 2: MG06185.1	109 aa
Sequence 3: FG10368.1	108 aa
Sequence 4: AN5719.1	109 aa
Alignment Length: 110 aa
Number Identitical Residues: 74 aa
Alignment Score (without introns) 3028


MG06185.1 	MSTSELATSYAALILADDGVEIT0SDKLQTLIKAAGVPDIEPIWAQLFAK0ALEGKDVKD
NCU08960.1	MSTAELATSYAALILADDGVEIT0ADKIQTIIKAAKIEDVEPIWASLFAK0ALEGKDVKD
FG10368.1 	MSHAELASSYAALILADDGIEIT0ADKLQTLIKAANVEEVEPIWTSIFAK0ALEGKDVKD
AN5719.1  	MSTAELACSYASLILADEGIEIT0ADKLQTLLTAAKVQEVEPIWTSIFAK~ALEGKDIKD
          	** :*** ***:*****:*:*** :**:**::.** : ::****:.:*** ******:**

MG06185.1 	LLSNVGSGGGAAAPAAGGAAAAGG-AAEEAKAEEKEEE1KEESDEDMGFGLFD
NCU08960.1	LLSNVGSGGAAAAPAAAGAAAAGG-AAEEAKAEEKVEE1KEESDEDMGFGLFD
FG10368.1 	LLVNVGSGGG-AAPAAGGAAAAGG-AEEAAPEEAKEEE1KEESDEDMGFGLFD
AN5719.1  	LLTNVGSAGA-AAPAGGAAPAAGGDAAAPAAEEKKEEE1KEESDEDMGFGLFD
          	** ****.*. ****...*.****.*   *  * * ** **************
```
